# The relationship between pain and athletic identity in youth ballet dancers

**DOI:** 10.3389/fpsyg.2025.1561450

**Published:** 2025-09-12

**Authors:** M. Minnat Choudhury, Emily Stapleton, Ashley Erdman, Emily Gale, Jane Chung, Sophia Ulman

**Affiliations:** ^1^Scottish Rite for Children, Dallas, TX, United States; ^2^University of Texas Southwestern Medical Center, Dallas, Texas, TX, United States

**Keywords:** athletic identity, ballet dancers, pain perception, adolescent psychology, identity formation

## Abstract

**Introduction:**

Ballet is considered both an art-form and a sport, intended to evoke an emotion through movement. Pain and the perception of pain is a part of ballet culture. Many adolescent ballet dancers begin training at a young age where musculoskeletal development is occurring congruently with psychological identity formation. Athletic identity is an identity facet defined as the degree an individual identifies with the athlete role. The relationship between pain and athletic identity during adolescence is currently unexplored.

**Methods:**

Forty-four American female ballet dancers between 10 and 18 years olds participated in a cross-sectional study in 2022 that involved completing surveys to understand the effect of pain on an athlete's relationship with dance. Dancers experiencing pain and dancers not experiencing pain were matched based on specialization level and age. Mann-Whitney U tests were performed to identify significant pain group differences in AIMS measures and dancer characteristics.

**Results:**

Dancers who reported experiencing pain had higher scores of athletic identity on measures of exclusivity and negative affectivity. Though all dancers trained more than 8 months out of the year without taking an offseason, dancers experiencing pain trained significantly more compared to dancers who were not experiencing pain, which may attribute to higher exclusivity identification.

**Discussion:**

These findings highlight the importance for dancers to reserve time in their training for rest and recovery, especially if taking an offseason is not possible. Additionally, dancer-specific education in coping with injury, pain, or poor performance is essential to support the emotional and physical well-being of dancers.

## Introduction

Ballet is both an art-form and a sport, revolving around evoking an emotional response from an audience through dance (Mcewen and Young, 2011; Jotwani et al., 2022). For adult dancers, pursuing dance with its daily routine of rehearsals, classes and performances can push dancers to push the boundaries of “the principles of human design” (Aalten, 2007). A result of this intensity is a high level of injuries (Blanchfield, 2016; Biernacki et al., 2021; Mattiussi et al., 2021). Given that ballet dancers frequently begin training in their youth during skeletal immaturity, the intensity of training may influence their musculoskeletal development, placing these dancers at risk for injury (Mcewen and Young, 2011; Blanchfield, 2016; Kolokythas et al., 2023). The culture behind ballet promotes rigorous time-intensive training, beginning with singular hours at a young age and progressing up to thousands of hours once professional (Jotwani et al., 2022). Classes can range between 1 to 4 h in length using repetitive movements involving the shoulders, spine, hips, knees and ankles, which may contribute to a risk of overuse injuries (Mcewen and Young, 2011).

### Coping with pain

One consequence of this high training volume and intensity is the high prevalence of pain reported among dancers (Blanchfield, 2016; De Souza et al., 2022; Paglione et al., 2023). Pain is almost inevitable in the continued pursuance and participation in dance, playing an important role in shaping and impacting dancers (Heil, 1999; Paglione et al., 2023). Ballet dancers have a complex relationship with dance that influences them emotionally, physiologically, physically, and mentally (Pickard, 2012). Pain, itself, is also complex, requiring dancers to psychologically adapt to it and to create coping mechanisms to manage it (Harrison and Ruddock-Hudson, 2017). However, to prioritize participation in dance, these coping mechanisms often involve making risky choices, potentially choosing to ignore injuries given the expectation of perseverance (Paglione et al., 2023).

The International Association for the Study of Pain (IASP) defines pain as “an unpleasant sensory and emotional experience associated with, or resembling that associated with, actual or potential tissue damage” (IASP, 2017). Pain can be described through a biopsychosocial approach as a multidimensional, dynamic interaction of factors, including psychological, physiological, and social factors, that influence one another (Meints and Edwards, 2018). One dimension of this is the sensory-discriminative dimension which refers to an intensity of pain including spatial and temporal characteristics along with quality of pain (Auvray et al., 2010). Another dimension is the affective-motivational dimension which refers to the level of “unpleasantness” the pain is, capturing the motivational aspect of pain which makes humans want to take protective action (Auvray et al., 2010).

### Athletic identity

For adolescent dancers, the self-construct of identity is also being formed during this time of psychological growth and musculoskeletal development (Pickard, 2012; Ohashi et al., 2023). While Erikson (1968) describes the self-construction of identity in *Identity: Youth and Crisis*, Markus (1977) defined specific identities as cognitive structures. Cognitive structures are used to guide incoming self-information from lived experiences through an organizational structure (Markus, 1977). A specific identity that may be tied to athletes, and thus ballet dancers, is athletic identity, which is defined as “the degree to which an individual identifies with the athlete role.” Recent systematic reviews and meta-analyses have expanded the understanding of athletic identity in youth athletes, identifying both positive and negative implications for well-being, performance, and injury outcomes (Edison et al., 2021; Lochbaum et al., 2022; Brewer and Chatterton, 2024). Therefore, evaluating athletic identity can provide greater context behind the psychological mindset of an athlete.

In addition, the self-determination theory offers a valuable lens for interpreting how athletic identity may influence the relationship between pain and participation in ballet. This theory emphasizes the role of intrinsic and extrinsic motivation in sustaining engagement in activities, suggesting that the fulfillment of basic psychological needs for autonomy, competence, and relatedness underpins self-motivated behavior (Deci and Ryan, 1985). Within this context, a strong athletic identity may act as a protective factor, fostering resilience and adaptive coping strategies that help dancers manage discomfort while preserving their connection to the sport. Conversely, it may also promote persistence in ways that increase vulnerability to physical harm, particularly if external pressures or perceived obligations outweigh internal cues for rest or recovery. Integrating these perspectives highlights the importance of situating athletic identity within a broader motivational framework, thereby deepening the interpretation of its role in shaping how adolescent dancers respond to pain and the demands of training.

### Linking pain and athletic identity

Previous studies have explored the psychological impacts of discomfort, pain, and injuries on dancers, describing how young dancers learn to reframe or suppress negative emotions for the sake of performance (Pickard, 2012). However, the interconnection of pain perception and athletic identity has not yet been analyzed, specifically in youth ballet dancers. Across a variety of competitive sports, strong athletic identities have been associated with increased sport participation (Lamont-Mills and Christensen, 2006) and risk for overtraining (Kentää et al., 2015; Hilliard et al., 2017), possibly contributing to pain presentations. Additionally, unforeseen events such as an injury have been shown to disrupt the psychological stability and identity of the athlete (Brewer and Chatterton, 2024), which could have detrimental effects to their recovery (Renton et al., 2021). Prior literature suggests a complex relationship between pain and athletic identity, with youth athletes exhibiting higher pain-related distress when negative affectivity was stronger, and lower pain-related avoidance when social identity was more prominent (Hsu et al., 2021). Given that ballet dancers frequently experience pain and often demonstrate tendencies toward overtraining, understanding how athletic identity relates to pain in this population is essential for informing both psychological support and injury prevention efforts.

The purpose of this study was to explore the potential association between athletic identity and pain in adolescent ballet dancers. We hypothesized that dancers experiencing active pain would report a greater degree of athletic identity compared to dancers without pain given the restriction pain may have on their participation in ballet and increased risk for overtraining impacting potential pain or injury experiences. A causal effect between increased athletic identity and perceived pain was not identifiable, but highlighting this potential relationship may provide a greater understanding on the role athletic identity has on ballet dancers and their risk for injury.

## Materials and methods

### Participants

Classically trained, American female ballet dancers between the ages of 10 and 18 years were asked to participate in a one-time testing session that involved completing surveys to understand the effect of pain on an athlete's relationship with their primary sport. A total of 44 female ballet dancers between the ages of 10.6 and 18.0 years (14.3 ± 1.8 years, pain group: 22 dancers) completed the necessary questionnaires and were included for analysis. Notably, 56 dancers were included in the parent study of which 34 reported experiencing pain. To create two even groups, dancers experiencing pain and dancers not experiencing pain were matched based on specialization level and age (±1 year). Thus, the pain group was narrowed down to match the no pain group which consisted of 22 dancers. Participants were excluded from enrollment in the study if they reported a recent musculoskeletal injury within the previous 3 months or were diagnosed with an orthopedic condition that would limit their ability to participate in ballet. No participants were lost or withdrawn from the study following enrollment. Although matching was employed to reduce baseline differences between groups, we acknowledge the potential for residual confounding due to unmeasured factors such as training intensity, coaching environment, or psychological characteristics. The study was approved by a regional institutional review board with all participants providing informed assent and/or consent prior to participation.

### Instruments

The specialization scale questions in the sports activity survey were used to establish the dancer's specialization level (Jayanthi et al., 2011, 2015). Participants answered either *yes* or *no* to each question which asked (1) “Did you quit other sports to participate in dance?”, (2) “Do you spend more than 8 months out of the year participating in dance?”, and (3) “Do you feel like you have a primary dance style?”. A zero value was assigned per “*no*” response and a value of 1 was assigned per “*yes*” response. The values from the three answers were summed to determine a numeric score. A total score of 0 or 1 indicated low specialization, 2 indicated moderate specialization, and 3 indicated high specialization. While reliability for this instrument has not been reported, numerous reports have utilized this questionnaire to classify specialization as it is currently the best option (Bell et al., 2016; Jayanthi et al., 2019; Okoruwa et al., 2022).

The 10 statement Athletic Identity Measurement Scale (AIMS) questionnaire, developed by Brewer et al. (1993), was administered to measure the degree of strength in which the participant identified with the athlete role or the degree in which one devotes special attention to sports relative to other engagements or activities in life. The questionnaire item responses ranged from 1 (strongly disagree) to 7 (strongly agree), utilizing a seven-point Likert scale to indicate agreement with each statement, such as “I consider myself an athlete.” The sum of values from all 10 statements in the questionnaire were totaled to determine the total AIMS score. Acceptable internal consistency has been reported (Cronback alpha = 0.81–0.93; Brewer et al., 1993; Good et al., 1993; Brewer and Cornelius, 2001). The original version of the AIMS was used without modification to maintain the validity of the instrument. This decision was based on the conceptualization of dancers as athletes and ballet as a sport, which aligns with prior research applying the AIMS to a mixed cohort of athletes (Hsu et al., 2021).

This total AIMS measure was further broken down into three subfactors of social identity, exclusivity, and negative affectivity. Briefly, (1) *social identity* identifies the strength at which an individual's self-worth is tied to their athletic identity, (2) *exclusivity* identifies the strength at which an individual is tied to the athlete role relative to other identities and roles, and (3) *negative affectivity* identifies the measure of the individual's emotional state affecting their athlete role from unwanted sporting outcomes (Brewer et al., 1993). Subscale scores range from 3 to 21 for social identity, 4 to 28 for exclusivity, and 2 to 14 for negative affectivity.

Participants were asked whether they were actively experiencing pain via the PROMIS Pain Intensity measures, which is a single question, “In the past 7 days, how bad was your pain on average” (Mara et al., 2023). Responses are captured on a scale from 0 (no pain) to 10 (worst pain you can think of). Participants who reported pain levels of 1 through 10, indicating some level of pain was experienced, were asked to complete two additional PROMIS pediatric framework surveys, developed by Mara et al. (2023), to understand the sensory and affective dimensions of the pain they were experiencing with raw scores recorded. The Pain Quality Sensory survey asks a series of 8 questions in an effort to qualitatively describe the pain the participant has experienced in the past 7 days along with the intensity of the pain. Participants answered via a Likert Scale from 1 (Not at all) to 5 (Very much) to whether their pain felt, for example, “Sharp?” or “Achy?”. The Pain Quality Affective survey asks a series of 9 questions to describe feeling behind the pain the participant has experienced in the past 7 days. Participants answered in a yes/no format to whether their pain was “Miserable?” or “Awful?”, for example, with a “Yes” response corresponding numerically to a 1 and a “No” response to a 0. This encapsulates the cognitive understanding of the experienced pain and the level of “unpleasantness”. From these surveys, the raw scores were converted to T-Score values using the short form conversion tables specific for a pediatric population provided by PROMIS. The Pain Quality Sensory and Pain Quality Affective surveys have demonstrated acceptable internal consistency, with Cronbach's alpha coefficients of 0.88 and 0.87, respectively (Mara et al., 2023).

### Procedures

Data collection for the current study occurred between May and November of 2022. Pre-professional ballet dancers were recruited for a larger parent study that involved the study team capturing a variety of functional tests at local ballet studios. In total, the study team visited four local studios in which adolescent female ballet dancers were recruited. As part of the larger parent study, participants were compensated for their participation. Once enrolled, participants completed a series of electronic surveys on a tablet device provided by the study team. All survey data were collected and managed using REDCap (Research Electronic Data Capture), a secure, web-based software platform designed to support data capture for research studies. Participants were specifically reassured that their responses were confidential and therefore not shared with their parents or dance instructors.

Participants first completed a survey indicating demographic information such as age and sex, as well as a custom sports activity participation survey developed to collect subject-reported outcomes (Appendix A). The sports activity participation survey included questions regarding the amount of time the athlete dedicated to their sport, the highest achieved competition level for their primary sport, and whether they identified as a multi- or single-sport athlete. In addition, the participation survey included the specialization scale questions, which were used to determine the athlete's exclusiveness and specialization in their self-identified primary sport (Jayanthi et al., 2013). The AIMS questionnaire was administered to quantify the individual's athletic identity (Brewer et al., 1993). Three surveys developed by the Patient-Reported Outcomes Measurement Information System (PROMIS) were administered to dancers experiencing pain the day of testing to understand pain intensity and quality. These surveys include the Pain Intensity survey, the Pain Quality Sensory survey and the Pain Quality Affective survey (Jacobson et al., 2015).

### Statistical analysis

Two groups were created based on the response to the initial pain intensity survey. Participants who responded with 0 were deemed as the “no pain” group and participants who responded with a non-zero value were deemed the “pain group”. Descriptive statistics for each pain group including frequencies, means and standard deviations for continuous measures (total and subscale scores), and medians and interquartile ranges (IQR) for ordinal measures (question/item scores) were calculated across all variables. Given significant Shapiro-Wilk tests of normality, non-parametric analyses were conducted between the pain groups. Specifically, Mann-Whitney U tests were performed to identify significant group differences in AIMS measures and dancer characteristics, including age, age at specialization, years dancing ballet, months per year dancing ballet, and specialization level. An effect size for each Mann-Whitney U test was calculated using the *z* statistic, with the rank-biserial correlation (*r*) providing a standardized measure of the magnitude of the group differences. 95% confidence intervals for the effect size were also computed to assess the precision of the estimates. Additionally, for the pain group, Spearman correlations were performed to identify significant associations between pain level reported and both AIMS measures and dancer characteristics. Statistical significance was concluded when *p* < 0.05. Statistical tests were conducted utilizing the IBM SPSS Statistics program.

## Results

The sample population of dancers consisted of 14% low, 64% moderate, and 23% highly specialized dancers. No significant group differences were found in age, years dancing ballet, years on pointe, age at specialization, practices per week, or specialization level (Table 1). The only significant difference in demographics or sport characteristics found between the two groups was in the number of months per year actively training for dance. The pain group reported training slightly more often [12.0 vs. 11.6 months, *p* = 0.01, *r* = −0.56 (−0.79, −0.18)], however, both groups indicated training nearly year-round. Additionally, for the pain group, no significant correlations between pain level and AIMS measures were found. A significant correlation was only identified between pain level and age (*r* = 0.48, *p* = 0.022), indicating older dancers reported higher levels of pain.

**Table 1:** Dancer demographics and sport characteristics.

**Variable**	**No pain Mean ±SD**	**Pain** **Mean ±SD**	***p***	***r***
Age (years)	14.2 ± 1.8	14.4 ± 1.8	0.460	−0.16 (−0.54, 0.28)
Years dancing ballet	10.2 ± 2.9	10.2 ± 3.0	0.869	−0.04 (−0.45, 0.39)
Years on pointe	3.2 ± 1.8	3.3 ± 2.1	0.905	−0.03 (−0.44, 0.40)
Age at specialization (years)	6.0 ± 4.5	6.1 ± 4.5	0.773	−0.06 (−0.47, 0.37)
Months per year dancing	11.6 ± 0.7	12.0 ± 0.2	**0.009**	−0.56 (−0.79, −0.18)
Practices per week	6.6 ± 1.5	6.2 ± 1.5	0.296	−0.22 (−0.59, 0.22)
**Specialization measures**	***N*** **(%)**	***N*** **(%)**
Quit non-dance sport(s) (% yes)	10 (45.5%)	12 (54.5%)
Train ≥ 8 months out of the year (% yes)	22 (100%)	22 (100%)
Ballet as primary dance style (% yes)	14 (63.6%)	12 (54.5%)

The bold p-values are to indicate statistical significance, which should be any value less than 0.050.

On a scale of 1–10, the level of pain indicated within the pain group ranged from 1 to 7, with an average of 3.8 ± 1.8. The average T-Score for Pain Quality Sensory was 46.7 ± 5.1 whereas the average T-Score for Pain Quality Affective was 43.9 ± 4.8 (Table 2). Both scores indicate that the dancers' sensory and affective pain qualities are slightly below the average of the reference population, suggesting they experience somewhat lower-than-average sensations and emotional responses related to pain. Of the pain group, 95% of these dancers indicated that their pain was a combination of multiple characteristics listed in the Quality Sensory survey with varying degrees of intensity per characteristic. Many of the dancers indicated their greatest intensity of pain was due to soreness (3.1 out of 5) compared to their lowest intensity of pain due to burning (1.2 out of 5). Many of the dancers in the pain group described the feeling behind their pain to be “unpleasant” and “annoying” per the Pain Quality Affective survey, whereas the least number of dancers described their pain as feeling “miserable”, “awful” and “horrible”. Pain was described as multifaceted by 77% of the dancers as indicated by selecting two or more characteristics to describe their pain.

**Table 2:** PROMIS pain quality sensory and affective.

**Pain Quality - Sensory**	**Mean ±SD**
In the past 7 days, did your pain feel…	
1. Sharp?	2.0 ± 0.8
2. Achy?	2.5 ± 1.1
3. Burning?	1.2 ± 0.7
4. Throbbing?	1.8 ± 0.9
5. Stabbing?	1.3 ± 0.5
6. Sore?	3.1 ± 1.4
7. Tight?	2.6 ± 1.4
8. Tingly?	1.5 ± 0.9
**Pain Quality - Affective**	***N*** **(%)**
In the past 7 days, did your pain feel…	
1. Miserable?	1 (9.1%)
2. Awful?	1 (4.5%)
3. Horrible?	1 (4.5%)
4. Unbearable?	1 (4.5%)
5. Worrying?	9 (40.9%)
6. Unending?	3 (13.6%)
7. Annoying?	17 (77.3%)
8. Unpleasant?	18 (81.8%)

The majority of dancers experiencing pain indicated the location of their pain being primarily at the ankle or hip, followed by the lower back and foot (Table 3). Additionally, most dancers indicated experiencing pain only in their lower extremities, but 27% indicated pain in both their upper and lower extremities at the time of testing. Pain was indicated in two upper extremity locations in 9% of dancers while 18% experienced pain in two locations in their lower extremity.

**Table 3:** Location of dancers' pain.

**Pain location**	***N* (%)**
Lower back	6 (27.3%)
Hip	8 (36.4%)
Knee	4 (18.2%)
Ankle	8 (36.4%)
Foot	6 (27.3%)
Other	4 (18.2%)
**Pain extremity**	***N (%)***
Upper	12 (54.5%)
Lower	14 (63.6%)
Both	6 (27.3%)

Responses sum greater than the number of participants given multiple pain locations and/or extremities could be selected.

In comparing athletic identity scores, the two groups demonstrated slightly but noteworthy differences (Table 4). Across all dancers, the average total AIMS score was 53.5 ± 9.2 out of 70. For dancers not experiencing pain, the average total AIMS score was 51.9 ± 6.9, whereas dancers experiencing pain reported an average total AIMS score of 55.1± 11.2 [*p* = 0.065, *r* = −0.39 (−0.70, 0.03)]. Although this difference did not reach statistical significance, it reflects a trend toward stronger athletic identity among dancers experiencing pain.

**Table 4:** AIMS scores and individual question responses for all participants and each pain group.

**AIMS scores**	**ALL**	**No pain**	**Pain**	***p***	***r***
Total AIMS	53.5 ± 9.2	51.9 ± 6.9	55.1 ± 11.2	0.065	−0.39 (−0.70, 0.03)
Social identity	19.2 ± 2.8	19.2 ± 2.3	19.1 ± 3.4	0.768	−0.06 (−0.47, 0.37)
Exclusivity	18.6 ± 4.3	17.6 ± 3.1	19.7 ± 5.2	**0.041**	−0.43 (−0.72, −0.02)
Negative affectivity	10.5 ± 2.5	9.9 ± 2.3	11.2 ± 2.7	**0.048**	−0.42 (−0.72, 0.00)
**AIMS questions**	**ALL**	**No pain**	**Pain**	***p***	***r***
1. I consider myself an athlete.	7.0 (1.0)	7.0 (1.0)	7.0 (1.0)	0.737	−0.07 (−0.48, 0.36)
2. I have many goals related to sport.	7.0 (1.0)	7.0 (1.0)	7.0 (1.0)	0.661	−0.09 (−0.50, 0.34)
3. Most of my friends are athletes.	7.0 (1.0)	6.5 (1.0)	7.0 (1.3)	0.306	−0.22 (−0.59, 0.22)
4. Sport is the most important part of my life.	6.0 (1.8)	5.0 (1.0)	6.0 (2.5)	0.260	−0.24 (−0.60, 0.20)
5. I spend more time thinking about sport than anything else.	5.0 (2.0)	5.0 (2.0)	5.0 (2.3)	0.772	−0.06 (−0.47, 0.37)
6. I need to participate in sport to feel good about myself.	5.0 (3.0)	4.0 (2.3)	6.5 (2.0)	**0.001**	−0.71 (−0.87, −0.41)
7. Other people see me mainly as an athlete.	5.0 (3.0)	5.0 (2.3)	5.5 (3.0)	0.904	−0.03 (−0.44, 0.40)
8. I feel bad about myself when I do poorly in sport.	5.0 (2.0)	5.0 (1.3)	6.0 (2.3)	**0.017**	−0.51 (−0.77, −0.11)
9. Sport is the only important thing in my life.	3.0 (3.0)	3.0 (3.3)	3.0 (3.3)	0.783	−0.06 (−0.47, 0.37)
10. I would be very depressed if I were injured and could not compete in sport.	6.0 (2.0)	5.0 (1.3)	6.0 (2.3)	0.328	−0.21 (−0.58, 0.23)

Total and subscale scores presented as Mean ± SD, and Question scores presented as Median (IQR). Subscale scores for each AIMS subscale range from 3–21 for social identity, 4–28 for exclusivity, and 2–14 for negative affectivity. The bold p-values are to indicate statistical significance, which should be any value less than 0.050.

Subscale analyses revealed more pronounced group differences. Dancers experiencing pain reported significantly higher exclusivity scores [*p* = 0.041, *r* = −0.43 (−0.72, −0.02)], indicating greater prioritization of sport participation over other aspects of life. Dancers in the pain group also reported significantly higher negative affectivity scores [*p* = 0.048, *r* = −0.42 (−0.72, 0.00)], suggesting more frequent negative emotions tied to poor performance or the inability to participate in their sport. In contrast, no significant differences were found in social identity scores [*p* = 0.768, *r* = −0.06 (−0.47, 0.37)], which implies comparable levels of self-concept as athletes between pain groups exists.

Median responses to each question of the AIMS across all dancers and between groups is also depicted in Table 4. Dancers experiencing pain related the least to statement 6 [*p* = 0.001, *r* = −0.71 (−0.87, −0.41), “I need to participate in sport to feel good about myself”], suggesting diminished emotional dependence on participation despite elevated overall identity scores. Alternatively, dancers experiencing pain related more to statement 8 [*p* = 0.017, *r* = −0.51 (−0.77, −0.11), “I feel bad about myself when I do poorly in sport”] compared to the non-pain cohort, reinforcing the observed elevations in negative affectivity. Scores for the other AIMS statements were similar between both groups. Overall, these findings suggest that while total athletic identity may not differ significantly by pain status, specific dimensions of that identity, particularly exclusivity and emotional investment, are heightened in dancers that report experiencing pain.

## Discussion

The purpose of the current study was to explore a potential connection between the presence of pain and athletic identity in youth ballet dancers. The hypothesis that dancers experiencing active pain would exhibit a greater degree of athletic identity compared to dancers not experiencing pain was partially supported. While total athletic identity scores did not significantly differ between dancers with and without pain, those experiencing pain demonstrated significantly higher exclusivity and negative affectivity, indicating greater prioritization of dance and stronger emotional responses to performance setbacks. These findings suggest that pain may be associated with more rigid or emotionally charged aspects of athletic identity, even in the absence of differences in overall identification as an athlete. Furthermore, while directionality was not investigated, the findings presented reinforce the idea that pain may not merely coexist with athletic identity but may be associated with differences in development. Specifically, repeated exposure to and tolerance of pain may be associated with higher exclusivity scores, reflecting a potential tendency for dancers to link commitment and toughness with self-worth in the athlete role. This interpretation aligns with recent systematic reviews and meta-analyses that describe athletic identity in youth athletes as a double-edged construct, capable of fostering motivation, persistence, and resilience, yet also increasing vulnerability to psychological distress and maladaptive behaviors when it becomes overly exclusive or is threatened by injury (Edison et al., 2021; Lochbaum et al., 2022; Brewer and Chatterton, 2024). In this context, the elevated exclusivity and negative affectivity observed in dancers experiencing pain may reflect a form of identity that sustains engagement but also heightens the risk of ignoring physical warning signs in order to preserve one's role in the dance environment.

Studies have reported that dancing at a high level can be associated with the experience of pain. Harrison and Ruddock-Hudson (2017) found that professional ballet dancers viewed pain as a subjective experience and could comprehend the difference between performance pain as “good pain” and injury pain as “bad pain”. The professional ballet dancers characterized bad pain as feeling “sharp”, “uncomfortable”, and “wrong” (Harrison and Ruddock-Hudson, 2017). Specifically, of the 20 participants, 19 admitted to pushing through pain to perform both in class and on stage (Harrison and Ruddock-Hudson, 2017), creating a normalized outcome of pain being seen as something to be expected and pushed through. Comparable to the current study, dancers who were experiencing active pain (i.e., experienced in the past 7 days) indicated dancing slightly more months during the year compared to their peers who did not indicate experiencing pain. Given dancers in pain reported year-round training habits, and that the current study only recruited dancers currently participating in dance training, it may be concluded that the dancers experiencing pain were failing to rest and recover, choosing to not take time off. Although the majority of dancers in the pain group described their pain as “unpleasant” and/or “annoying”, pain was most commonly related to soreness, implying a potentially similar perception of “good pain” as reported in prior studies. Regardless, the high prevalence of reported soreness further emphasizes the need for these dancers to incorporate adequate rest and recovery into their training regimen, especially if an offseason is not possible.

This level of dedication and drive to push through pain may be partly accounted for by the dancers' reported exclusivity scores, or the strength at which an individual is tied to the athlete, or dancer, role. In the pain group, exclusivity scores were significantly elevated, indicating a greater identification with being a dancer relative to any other identity or role they have assumed in their life. However, it is also possible that certain personality traits, such as perfectionism or achievement orientation, may predispose dancers to both stronger athletic identities and a greater likelihood of tolerating pain, thus shaping their responses to identity-related survey items. Nevertheless, pain is likely seen as simply an unavoidable facet of being a dancer and is potentially not enough of a hindrance to compel a dancer to take much needed rest. Instead, this intensification of the dancer identity may be related to chronic or recurring pain experiences, in which dancers come to view pain endurance as evidence of their legitimacy and belonging within the dance world. Pain may therefore be interpreted as both a reflection of their identity as dancers and a perceived requirement for maintaining that identity.

For many dancers, the acceptance of pain is allowable due to their love of the sport, but also, many dancers believe pain is required to achieve their career goals (Mcewen and Young, 2011). The experience of pain is perceived not as a barrier to continuing dance, but rather an indication of hard work and becoming better as dancers identify pain as an optimistic feeling (Wainwright and Turner, 2003; Aalten, 2007; Mcewen and Young, 2011; Markula, 2015). From a motivational perspective, this pattern may reflect both intrinsic and extrinsic factors. Intrinsic motivation, such as love of dance and the satisfaction of personal growth, may help dancers accept pain as part of progress. Alternatively, extrinsic motivation, such as pleasing instructors or competing for roles, may increase the likelihood of ignoring pain when external rewards are at stake. This balance aligns with the self-determination theory, which suggests that the interaction between internal motives and external pressures shapes whether pain is interpreted as constructive or harmful (Deci and Ryan, 1985).

Unfortunately, given this perception of dancers and their drive for excellence, it may only be their instructors that have the power to alter this narrative. Youth dancers have been reported to hyperfocus on pleasing their instructor to achieve selective performance opportunities, which may include adopting certain values, behaviors, and expectations (Pickard, 2012). Instructors serve as both gatekeepers to advancement and role models for acceptable behavior, and therefore, their attitudes toward pain, rest, and performance heavily influence dancers' beliefs and decision-making. If instructors implicitly or explicitly reinforce the idea that pushing through pain is admirable, or even expected, young dancers may internalize these messages as essential to success. Conversely, when instructors promote balanced training, normalize rest, and model healthy responses to injury, they can help shape a more sustainable and psychologically supportive culture. Future work is needed to empower instructors to create healthier environments in support of their dancers.

Several studies have also shown that dancers embody a great need to remain competitive amongst their peers (Wainwright and Turner, 2003; Wainwright et al., 2005; Mcewen and Young, 2011; Markula, 2015). However, alongside competitiveness, the inability to recognize serious injuries, including those that stem from pain, can jeopardize a dancer's performance and/or career (Aalten, 2007; Tarr and Thomas, 2011; Harrison and Ruddock-Hudson, 2017; Pollard-Smith and Thomson, 2017). Wainwright and Turner (2006) found that professional dancers may perceive the consequence of missing a performance due to pain or injury to be a detriment to their career, which may be further exacerbated by the short nature of a dance career. Mcewen and Young (2011) and Wainwright et al. (2005) both found that professional dancers connected the perception of lost time performing to a potential loss of identity, which in turn may negatively impact a dancer's performance or career.

This perception may be present or influenced from a young age. In the current study, significant differences were found in negative affectivity, specifically in statement 8 (“I feel bad about myself when I do poorly in sport”). The biopsychosocial model of pain indicates that negative mood impacts pain perception and makes it harder to cope. An alternative explanation may be that dancers with higher trait neuroticism or sensitivity to evaluation are more prone to negative affect, which in turn may amplify their experience of pain and increase their emotional investment in performance. Intrinsic motives rooted in mastery and enjoyment may buffer against the emotional toll of pain, while extrinsic motives tied to approval or opportunities may amplify its negative impact. This highlights the importance of considering both motivational context and identity when evaluating how dancers respond to pain.

Dancer-specific education in coping with injury, pain, or poor performance is essential for the emotional and physical well-being of dancers. Additionally, the integration of pain into the dancer's identity may influence emotional regulation strategies and coping mechanisms, particularly during adolescence when identity development is highly malleable. Without appropriate support, dancers may internalize pain as a normative and even necessary part of performance, potentially leading to maladaptive psychological outcomes such as perfectionism, burnout, or injury denial. Future interventions should consider not only physical recovery, but also the cognitive and emotional restructuring of identity in relation to pain. Incorporating motivational strategies, such as promoting intrinsic goal setting, autonomy supportive coaching, and balanced performance standards, may reduce reliance on external reinforcements that contribute to unhealthy persistence when experiencing pain. Embedding preventive frameworks and psychological skills programs within training environments, such as goal-setting workshops, guided recovery planning, and emotional regulation training, may further help dancers reframe their relationship with pain while maintaining performance quality.

Similarly, while not found to be significant, dancers experiencing pain related greater to being depressed if injured and thus unable to compete. Given the level of specialization, effort and training presented in the youth ballet dancers in the present study, the perception of lost time due to an injury is similar to that described in professional ballet dancers from previous studies. This may, in turn, affect the mental and emotional psychological development during adolescence. Interestingly, even though exclusivity was overall elevated in the pain group, one contributing question to the exclusivity subfactor was notably greater in the no pain group—statement 6 (“I need to participate in sport to feel good about myself”).

This study has limitations to note. While the presented findings highlight a relationship between the psychological perception of pain and athletic identity, the cross-sectional nature of the study precludes any conclusions regarding the causality of this relationship. The relatively small and homogenous sample, composed exclusively of American female ballet dancers, may also limit generalizability of findings. While the authors believe the sample group presented in this study reflects the population of female youth ballet dancers well (Downing et al., 2022; Pruš and Zaletel, 2022), cultural, sex, or training context differences, as well as parental influence or competitive environment, may shape both pain perception and athletic identity. As such, the results should be interpreted with caution and applied primarily within the context of pre-professional youth ballet settings, rather than broadly to all adolescent athletes. Additionally, classification into the pain and no pain groups relied on a single item, which may overlook nuances such as pain duration, frequency, or context. Finally, while the AIMS was administered in its original form to preserve the instrument's validated structure, we acknowledge that its language may not fully capture the unique experiences of ballet dancers. These limitations specific to both the pain intensity question and AIMS could have influenced how some participants interpreted and responded to certain items. Future studies may consider a more complex method for classifying dancers into a pain group, as well as adapting or supplementing the AIMS to better reflect the identity constructs relevant to dance populations. Despite these limitations, this study is among the first to empirically examine the intersection of pain and athletic identity in youth ballet dancers, a population often underrepresented in sport psychology research. The use of validated instruments and the focus on a well-defined, pre-professional cohort enhances the relevance of the findings to high-level dance training environments.

The current findings provide important implications for both research and practice. Longitudinal studies are needed to determine whether the relationship between pain and athletic identity is directional or bidirectional, and how these dynamics evolve across stages of dance training. Future research should also examine more diverse populations including dancers of different cultural backgrounds, competition levels, and dance styles, and consider adapting the AIMS to be specific for dance populations. Incorporating qualitative data in future studies could further enrich the interpretation of these results, providing deeper insight into the lived experiences of dancers. Focus groups or interviews with dancers, instructors, and clinicians may offer valuable perspectives on the psychological impacts of pain and athletic identity that quantitative measures alone cannot fully capture. Furthermore, the findings presented in this study underscore the importance of integrating psychological support into dance training environments. Clinicians and instructors may benefit from screening for athletic identity levels and pain status to better understand a dancer's psychological investment and potential vulnerability. Early identification of maladaptive identity traits such as excessive exclusivity or negative affectivity may allow for targeted interventions that promote psychological well-being and sustainable participation in dance. Broadly, acknowledging the psychological dimensions of pain may help shift the culture in youth ballet toward one that values both performance and mental health.

## Conclusions

In conclusion, dancers who reported experiencing pain displayed stronger identification with negative affectivity and exclusivity compared to their peers who were not experiencing pain. Though all dancers train more than 8 months out of the year without taking an offseason, dancers experiencing pain trained significantly more months out of the year compared to dancer who were not experiencing pain, which may attribute to higher exclusivity identification. These findings emphasize the need for dancers to reserve time in their training for rest and recovery, especially if taking an offseason is not possible. Future work should investigate the perception of pain and athletic identity over time to better understand how pain may influence the formation of athletic identity in ballet dancers and vice versa. Additionally, research is needed to develop and disseminate dancer-specific education in coping with injury, pain, or poor performance to support the emotional and physical well-being of dancers.

## Data Availability

The raw data supporting the conclusions of this article will be made available by the authors, without undue reservation.

## Appendices

**Appendix A**

1. How many years have you participated in dance?
2. How many years have you danced en pointe?
3. How many months per year do you participate in dance?
4. How many times do you practice per week for at least an hour?
5. What styles of dance do you train in other than ballet?
6. Do you feel like you have a primary dance style?
7. What is your primary dance style?
8. Do you spend more than 8 months out of the year participating in dance?
9. Did you quit other sports to participate in dance?
10. Do you take an offseason from dance?
11. Do you play other sports competitively? a. If yes, what age did you decide to only participate in dance? b. If no, what other sports do you play competitively?
12. Have you ever had an activity related injury?
13. What year(s) or what grade(s) did your injury occur?
14. Where was your injury?
15. Have you had any activity-related injuries in the last year?
16. How many activity-related injuries have you had in the last year?
